# In vitro evaluation of forages and feedstuffs from Kenya’s semi-intensive zero-grazing dairy system

**DOI:** 10.3168/jdsc.2025-1005

**Published:** 2026-05-15

**Authors:** Edward H. Cabezas-Garcia, Yang Li, Daniel Korir, Jesse K. Gakige, Stefan Muetzel, César S. Pinares-Patiño, Vibeke Lind, Mutian Niu, Claudia Arndt

**Affiliations:** 1 International Livestock Research Institute (ILRI), Nairobi 00100, Kenya; 2 Animal Nutrition, Institute of Agricultural Sciences, Department of Environmental Systems Science, ETH Zürich, 8092 Zürich, Switzerland; 3 Department of Animal Science, University of New England, 2351, Armidale, New South Wales, Australia; 4 AgResearch Ltd. Grasslands, Palmerston North, 4442, New Zealand; 5 Norwegian Institute of Bioeconomy Research (NIBIO), NO-1431 Ås, Norway

## Abstract

This study evaluated 9 alternative forages at a realistic 12% of dietary DM within semi-intensive zero-grazing Kenyan dairy cow on in vitro gas production and rumen fermentation parameters. Incubation substrates contained a common basal feed mixture plus either *Lolium multiflorum* (control treatment) or *Medicago sativa*, *Vicia sativa*, *Desmodium intortum*, *Calliandra calothyrsus*, *Leucaena leucocephala*, *Sesbania sesban*, *Tradescantia zebrina*, *Cichorium intybus*, or *Ipomoea batatas*. Methane production was analyzed with mixed models and its marginal relationships with tannins fractions and nutritional composition with generalized additive models. Results showed that *Calliandra calothyrsus* and *Leucaena leucocephala* decreased methane production by 10% to 13% without impairing total gas, digestibility, major VFA profiles, or estimated metabolizable energy. *Desmodium uncinatum* and *Tradescantia zebrina* slightly shifted fermentation toward higher propionate, branched-chain VFA, and ammonia, as well as lower pH, whereas *Vicia sativa* reduced total VFA concentration. Condensed tannins and starch were negatively, and organic matter digestibility positively, associated with methane production. The inclusion level of *Calliandra calothyrsus* and *Leucaena leucocephala* legumes tested here demonstrates promising potential for practical incorporation into semi-intensive zero-grazing dairy cattle diets to mitigate enteric methane in Kenyan dairy systems.

## Highlights

• *Calliandra* and *Leucaena* lowered methane emissions in vitro at 12% diet inclusion.
• Condensed tannins were the strongest diet factor linked to lower methane.
• In vivo studies are needed to validate the potential of incubated feed mixtures.

## Graphical Abstract

**Figure:**
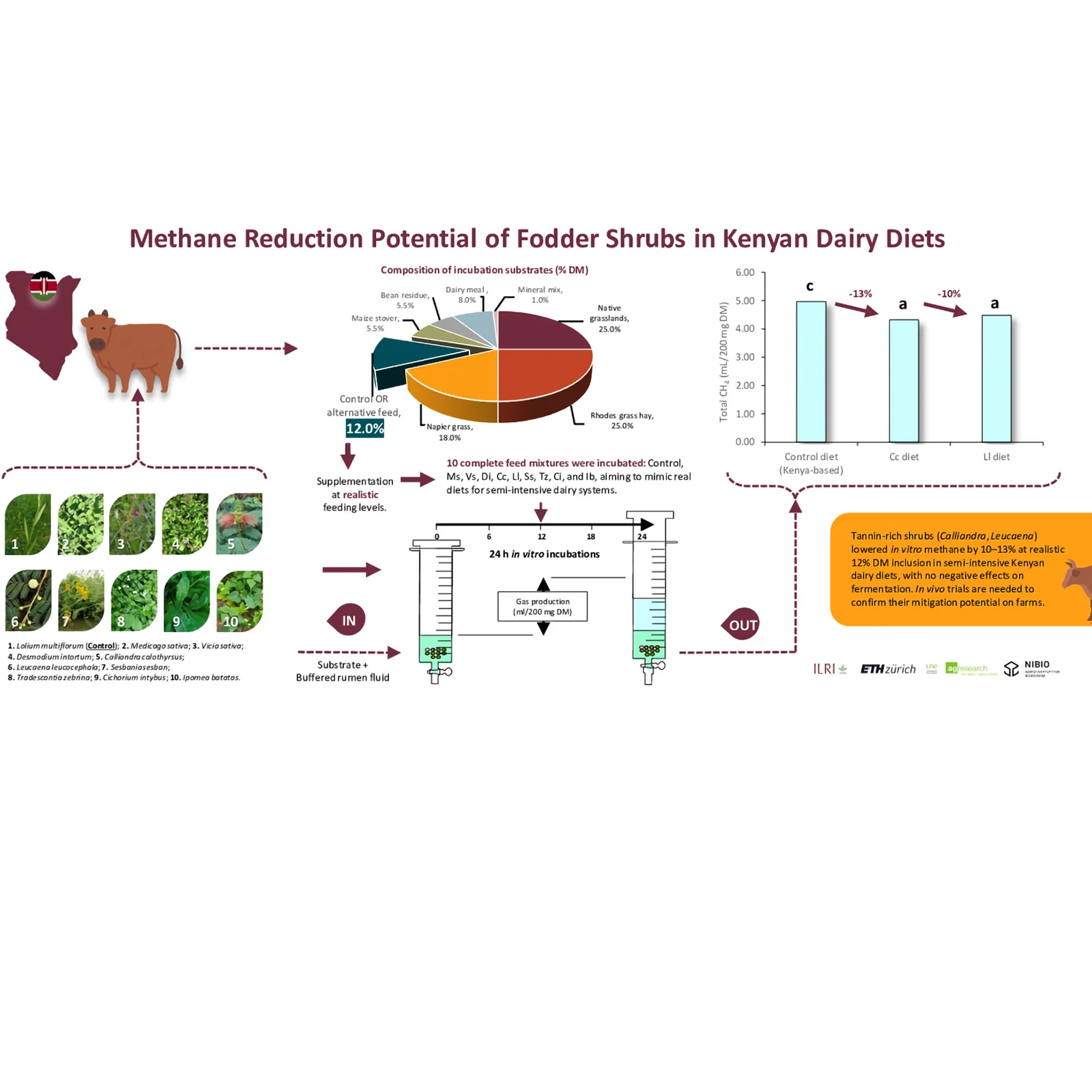
**Summary:** This in vitro study evaluated the effect of including locally available forages at 12% on a dry matter (DM) basis as part of typical semi-intensive zero-grazing dairy cow diets in Kenya. Incubated feed mixtures were formulated with either annual ryegrass (control) or 9 alternative forage species, including 6 legumes, 2 herbs, and 1 root-crop foliage, together with typical basal ingredients. Gas and methane production were measured after 24-hour incubations with buffered rumen fluid to simulate rumen fermentation. Feed mixtures containing *Leucaena leucocephala* or *Calliandra calothyrsus* reduced methane production by 10% to 13% compared with the control diet, without impairing substrate fermentability. These results indicate that tannin-containing fodder shrubs, already common on smallholder farms, may offer a practical approach to lowering enteric methane emissions when included at realistic supplementation levels. Further in vivo validation is necessary to confirm these findings in real-world dairy systems. Cc = *Calliandra calothyrsus*; Ci = *Cichorium intybus*; Di = *Desmodium intortum*; Ib = *Ipomoea batatas*; Ll = *Leucaena leucocephala*; Ms = *Medicago sativa*; Ss = *Sesbania sesban*; Tz = *Tradescantia zebrina*.

Cattle production is expected to expand in low- and middle-income countries, where agriculture remains a key livelihood (Adesogan et al., 2025). Improving feed efficiency while lowering environmental impacts is essential for sustainable growth (Godfray et al., 2010). Kenya's Tier-2 GHG inventory indicates that 40% to 45% of the national dairy herd is kept in semi-intensive systems (SDL, 2020). Dairy cattle emit ∼12.3 million tonnes of CO_2_ equivalents annually, with 88% as enteric methane (CH_4_; FAO and NZAGRC, 2017).

Enteric CH_4_ arises from rumen fermentation of dietary carbohydrates and represents a 2% to 12% loss of gross energy (Johnson and Johnson, 1995). Mitigation strategies such as improving diet quality, increasing feeding level, reducing forage maturity, or adjusting the forage-to-concentrate ratio can reduce CH_4_ intensity while improving productivity (Arndt et al., 2022). In Kenya, only interventions that both enhance productivity and reduce CH_4_ are feasible. For example, replacing low-CP, high-NDF forages with higher-quality alternatives can increase milk yield and lower CH_4_ per unit product, with possible added suppression from secondary plant compounds (Muia, 2000). Affordable, farm-available options are therefore needed. Leguminous fodder trees, shrubs, herbs, and root foliage, already common on semi-intensive farms, may improve rumen fermentation, animal performance, and CH_4_ mitigation while also providing added system benefits such as nutrient recycling and shade (Gulwa et al., 2018).

This study used an in vitro system to evaluate forages representative of semi-intensive zero-grazing diets (SDL, 2020). The assay assessed the effects of including annual ryegrass (*Lolium multiflorum*; control) or 9 alternative forages at 12% dietary DM in a basal feed mixture on total gas production, CH_4_ production, and rumen fermentation parameters. Annual ryegrass, a cool-season C3 forage introduced to supplement tropical pastures, served as a high-quality benchmark for assessing the methane-mitigation potential of locally available feedstuffs in Kenyan dairy systems.

In vitro incubations followed the Hohenheim Gas Test (Menke and Steingass, 1988). Rumen fluid was collected before morning feeding from 3 mid-lactation, rumen-cannulated Brown Swiss cows (706 ± 61 kg BW) housed at AgroVet-Strickhof (Lindau, Switzerland; license ZH115/2022). Donor cows were fed a TMR containing (DM basis) 52.8% grass silage, 14.1% corn silage, 14.1% ensiled sugar beet pulp, 11.4% concentrate, 6.8% hay, and 0.9% minerals (9.9% CP; 42.1% NDF; 21.7% ADF; 6.8% starch). Rumen fluid from each cow served as a biological replicate. Three syringes per treatment were incubated per cow and run, and each included 10 treatments plus 3 blanks. In total, 3 in vitro runs were carried out.

Rumen fluid was filtered through cheesecloth, kept at 39°C, and mixed with Menke buffer (1:2 vol/vol) under CO_2_. Glass syringes were loaded with 200 mg of diet DM and 30 mL of buffered rumen fluid, randomized by treatment, and incubated at 39°C for 24 h in a rotating incubator. Each run included 3 blanks. After incubation, total gas was recorded and headspace CH_4_, H_2_, and CO_2_ quantified by gas chromatography (thermal conductivity detector), following Soliva and Hess (2007). Net gas and CH_4_ were calculated by subtracting blanks. Buffered rumen fluid pH and ammonia were measured within 5 min of opening, VFA by HPLC, and OM digestibility (**OMD**) and ME were estimated according to Menke and Steingass (1988).

The basal feed mixture used to formulate the incubation substrate was designed to reflect semi-intensive zero-grazing Kenyan dairy systems (SDL, 2020) and, on a DM basis, comprised 25% native grassland, 25% Rhodes hay, 18% Napier grass, 5.5% maize stover, 5.5% bean residue, 8% dairy meal, and 1% minerals, totaling 88%. The remaining 12% of DM represented a realistic level of secondary forage supplementation and consisted of either *Lolium multiflorum* (control [**CONT**]) or one of 9 alternative forages: temperate legumes (*Medicago sativa* [**Ms**], *Vicia sativa* [**Vs**]), tropical legumes (*Desmodium intortum* [**Di**], *Calliandra calothyrsus* [**Cc**], *Leucaena leucocephala* [**Ll**], *Sesbania sesban* [**Ss**]), herbs (*Tradescantia zebrina* [**Tz**], *Cichorium intybus* [**Ci**]), or root foliage (*Ipomoea batatas* [**Ib**]). All ingredients were dried at 60°C for 48 h, ground to 1 mm, and stored in sealed jars. Unless otherwise stated, chemical composition analyses followed AOAC (1997). Dry matter and OM were determined gravimetrically; NDF and ADF (ash-free) via Fibertec System (FOSS Analytical, Hillerød, Denmark) following Van Soest et al. (1991); ether extract (**EE**) by Soxhlet extraction; CP by C/N analyzer (CP = N × 6.25); starch spectrophotometrically (Smith and Zeeman, 2006), and tannins according to Makkar (2003).

Data were analyzed via a mixed-effect model using the lmer procedure (Bates et al., 2015) using R (version 4.4.1; R Core Team, 2024). The model is as follows:

*Y_ijk_* = *µ* + *T_i_* + *C_j_* + *e_ijk_*,

where *Y_ijk_* is the variable of interest, *μ* is the overall mean, *T_i_* is the treatment effect of incubated feed mixture (*i* = 1 to 10), *C_j_* is the random effect of donor cow (*j* = 1, . . ., *J*), and *e_ijk_* is the residual error for observation *k* within the *i*, *j* combination (Li et al., 2024). Observations with studentized residuals >|3| were removed. Pairwise comparisons used Tukey's test. In vitro CH_4_ production was analyzed using a generalized additive mixed model (**GAM**) with tannin fractions, CP, NDF, starch, EE, and OMD as feed mixture covariates and cow included as a random effect. “Feed mixture-only” and “cow-only” additional GAM were also fitted to partition the variance, with the cow effect represented as a separate variance component from the residual error. Response curves from the full GAM were used to characterize the partial relationships between each dietary predictor and CH_4_ production. Smoothing terms and variable effects of the fitted GAM were estimated and visualized using the mgcv and mgcViz packages in R (Wood and Wood, 2015; Fasiolo et al., 2020). Significance was declared at *P* ≤ 0.05 and trends at 0.05 < *P* ≤ 0.10.

The nutritional composition and tannin contents of the individual ingredients and feed mixtures are presented in Table 1. Dietary CP of the incubated feed mixtures averaged 11.0% ± 0.63% DM, ranging from 12.1% (Ms) to 10.3% (Tz). The NDF fraction ranged from 51.9% (Ll) to 58.1% (Di) and ADF from 30.0% (Ci) to 35.3% (Di). Ether extract varied from 1.36% (Ms) to 1.73% (Ib), and starch ranged from 1.46% (Ll) to 2.52% (Ss). Total tannin concentration was greatest in Cc (0.91% DM) and Ll (0.85% DM), approximately 5-fold higher than in the remaining feed mixtures. Compared with Ll, the Cc treatment contained more hydrolyzable tannins (**HT**; 0.57% vs. 0.34% DM) and less condensed tannins (**CT**; 0.28% vs. 0.57% of DM). *Sesbania sesban* had the lowest total tannins (**TT**; 0.09% DM).

**Table 1:** Chemical composition of feed mixtures (treatments) and individual ingredients used for performing the in vitro incubations

Item	Chemical composition^1^									
	DM	OM	CP	NDF	ADF	Starch	EE	TT	HT	CT
Treatment^2^										
CONT	92.3	80.8	10.3	56.3	31.0	1.70	1.56	0.14	0.06	0.08
Ms	92.2	80.5	11.7	54.4	31.5	1.53	1.36	0.14	0.06	0.08
Vs	92.1	81.2	12.1	54.4	32.3	1.70	1.51	0.16	0.09	0.07
Di	92.2	80.7	10.7	58.1	35.3	2.51	1.54	0.16	0.03	0.13
Cc	92.0	80.7	10.5	56.0	33.1	2.09	1.57	0.91	0.57	0.34
Ll	92.2	81.3	10.7	51.9	30.5	1.46	1.67	0.85	0.28	0.57
Ss	91.9	81.0	11.2	54.2	31.4	2.52	1.58	0.09	0.01	0.09
Tz	91.9	79.7	10.3	54.9	31.5	1.76	1.67	0.15	0.04	0.10
Ci	92.0	80.0	11.5	53.6	30.0	1.61	1.58	0.14	0.05	0.09
Ib	91.8	79.3	10.7	53.8	30.7	2.25	1.73	0.29	0.22	0.07
Ingredients within the basal feed mixture^3^										
Native grassland forage	92.2	83.1	18.0	57.5	28.4	0.30	1.02	0.42	0.33	0.09
Rhodes grass hay	93.8	84.9	4.8	63.5	37.1	0.25	1.61	0.31	0.23	0.09
Napier grass	91.4	77.4	8.1	57.3	33.9	0.22	1.03	0.29	0.26	0.04
Maize stover	92.5	86.9	6.5	57.5	34.0	2.03	0.88	0.21	0.18	0.03
Bean residue	92.4	86.4	5.9	56.1	44.5	0.53	0.67	0.18	0.13	0.05
Dairy meal	91.4	71.4	9.2	31.2	18.7	36.5	3.93	0.02	0.01	0.01
Mineral mix	99.0	—	—	—	—	—	—	—	—	—
Forages within the individual feed mixtures^4^										
*Lolium multiflorum*	91.4	78.0	15.7	27.4	27.2	0.24	2.01	0.08	0.01	0.07
*Medicago sativa*	90.5	79.9	23.9	27.8	22.9	1.21	1.03	0.2	0.12	0.08
*Vicia sativa*	90.6	79.7	30.9	20.6	18.7	1.07	1.53	0.09	0.01	0.08
*Desmodium intortum*	90.2	82.8	17.3	47.8	37.6	0.64	0.99	0.63	0.20	0.43
*Calliandra calothyrsus*	89.2	84.0	16.5	29.0	12.4	7.01	1.51	8.46	6.17	2.30
*Leucaena leucocephala*	92.0	82.6	22.8	13.9	12.4	0.35	2.42	10.2	7.48	2.77
*Sesbania sesban*	89.0	82.9	22.7	19.0	12.0	23.0	1.71	0.45	0.03	0.42
*Tradescantia zebrina*	90.0	71.4	15.8	23.0	19.1	2.58	1.28	0.23	0.02	0.22
*Cichorium intybus*	89.4	72.0	20.4	23.6	12.5	0.47	2.57	0.11	0.04	0.07
*Ipomea batatas*	88.7	74.9	23.5	25.2	16.6	1.43	1.73	0.24	0.20	0.03

1 Except for DM (% as fed), all values are expressed as % of DM. EE = ether extract; TT = total tannins; HT = hydrolyzable tannins; CT = condensed tannins.

2 Ten feed mixtures containing *Lolium multiflorum* (CONT), *Medicago sativa* (Ms), *Vicia sativa* (Vs), *Desmodium intortum* (Di), *Calliandra calothyrsus* (Cc), *Leucaena leucocephala* (Ll), *Sesbania sesban* (Ss),*Tradescantia zebrina* (Tz), *Cichorium intybus* (Ci), or *Ipomoea batatas* (Ib) at the inclusion level of 12% on a DM basis.

3 Same feeds and inclusion levels were used within the basal feed mixture on a DM basis.

4 Scientific names for the different individual forages included within incubated feed mixtures.

*Calliandra calothyrsus* and *Leucaena leucocephala* are widely used in East Africa as cost-effective protein supplements (Roothaert and Paterson, 1997). At the realistic inclusion level of 12% DM evaluated here, tannin concentrations are all below thresholds associated with impaired nutrient use in vivo, consistent with earlier reports (Garcia et al., 1996; Mwangi et al., 2024). Higher inclusion rates (>23% DM) can depress digestibility in goats (Hove et al., 2001), but such levels exceed typical semi-intensive supplementation practices.

In vitro fermentation characteristics, digestibility, and estimated ME are presented in Table 2. Total gas production ranged from 43.0 (Cc) to 47.1 mL/200 mg DM (Ss), with no difference among treatments (*P* ≥ 0.05). Hydrogen production increased as pH decreased (*P* < 0.01), whereas CO_2_ was unaffected (*P* = 0.17). Methane ranged from 4.32 to 4.97 mL/200 mg DM, with CONT highest and Cc and Ll lowest (*P* ≤ 0.05), consistent with the observed partial inhibition of methanogenesis by CT without major effects on rumen fermentation (Huyen et al., 2016). *Calliandra calothyrsus* reduced CH_4_ production by 13% relative to CONT, consistent with reports that *Calliandra calothyrsus* tannins can lower methanogenesis in tropical grass-based diets with molasses supplementation (Hess et al., 2004). *Leucaena leucocephala* reduced CH_4_ by 10% compared with CONT (*P* < 0.01).

**Table 2:** The effect of evaluated feed mixtures (treatments) on total gas production, fermentation profile, and estimated digestibility and energy contents after 24-h in vitro incubation

Item	Treatment^1^										SE	*P-*value
	CONT	Ms	Vs	Di	Cc	Ll	Ss	Tz	Ci	Ib		
Gas production (mL/200 mg DM)												
Total gas	45.7^ab^	44.8^ab^	46.1^b^	44.3^ab^	43.0^a^	46.0^b^	47.1^b^	45.2^ab^	45.7^ab^	45.4^ab^	0.98	<0.01
H_2_	0.00133abc	0.00144abc	0.00233^c^	0.00436^de^	0.00373^d^	0.00416^de^	0.00199^bc^	0.00503^e^	0.00103^ab^	0.00056^a^	0.000558	<0.01
CO_2_	29.6	28.2	28.7	29.1	27.5	29.8	29.6	29.0	29.7	29.2	0.84	0.17
CH_4_	4.97^c^	4.73abc	4.75abc	4.55abc	4.32^a^	4.48^ab^	4.70abc	4.73abc	4.77^bc^	4.63abc	0.296	<0.01
CH_4_:CO_2_ ratio	0.169	0.163	0.162	0.158	0.160	0.151	0.160	0.167	0.162	0.159	0.0112	0.12
CH_4_/dOM^2^	45.4^b^	43.5^ab^	43.1^ab^	42.0^a^	41.5^a^	40.7^a^	42.1^a^	43.1^ab^	43.6^ab^	42.8^ab^	2.28	<0.01
In vitro fermentation profile												
pH	7.01^ef^	7.08^g^	7.00^de^	6.92^ab^	6.94^bc^	6.96bcd	6.96^cd^	6.89^a^	7.04^fg^	7.07^g^	0.055	<0.01
Total VFA (m*M*)	48.6^bc^	47.9abc	45.8^a^	47.0^ab^	47.1abc	48.3^bc^	47.3abc	47.0^ab^	49.4^c^	46.7^ab^	1.35	<0.01
Acetate (molar %)	71.2^bc^	69.8^ab^	72.5^c^	69.9^ab^	71.4^bc^	70.8abc	72.2^c^	69.1^a^	71.4^bc^	70.4^ab^	1.56	<0.01
Propionate (molar %)	17.1^a^	17.5^ab^	17.0^a^	18.4^bc^	17.6^ab^	17.1^a^	17.4^ab^	19.0^c^	16.9^a^	17.7^ab^	0.93	<0.01
Butyrate (molar %)	7.32^ab^	7.73^bc^	7.72^bc^	8.26^cd^	7.90bcd	7.05^a^	7.63abc	8.50^d^	7.69^bc^	7.55^ab^	0.554	<0.01
Isobutyrate (molar %)	1.72^bc^	2.12^c^	0.966^a^	1.12^a^	1.15^ab^	0.907^a^	0.958^a^	1.27^ab^	2.03^c^	2.02^c^	0.166	<0.01
Valerate (molar %)	0.515^a^	0.759^d^	0.549^a^	0.743^cd^	0.628abcd	0.611abc	0.533^a^	0.706bcd	0.589^ab^	0.639abcd	0.0598	<0.01
Isovalerate (molar %)	1.16^a^	1.75^d^	1.23^ab^	1.55bcd	1.33abc	3.77^e^	1.23^ab^	1.65^cd^	1.50abcd	1.75^d^	0.165	<0.01
A:P ratio	4.02^ab^	3.97^ab^	4.27^b^	3.81^ab^	4.08^ab^	4.15^ab^	4.17^ab^	3.71^a^	3.99^ab^	4.04^ab^	0.301	<0.01
NH_3_ (m*M*)	10.6^a^	11.2abc	11.4^bc^	11.4^bc^	10.5^a^	10.5^a^	10.6^a^	11.7^c^	10.8^ab^	11.0^ab^	0.60	<0.01
OMD^3^ (%)	60.9abc	60.7abc	62.0^bc^	59.8^ab^	58.6^a^	61.3^bc^	62.5^c^	60.5abc	61.4^bc^	60.8abc	0.82	<0.01
ME^3^ (MJ/kg DM)	8.42^ab^	8.51^ab^	8.62^ab^	8.34^a^	8.33^a^	8.65^ab^	8.72^b^	8.42^ab^	8.64^ab^	8.53^ab^	0.115	<0.01

a–g Within a row, means without a common superscript letter differ significantly at *P* < 0.05.

1 Ten feed mixtures containing *Lolium multiflorum* (control treatment [CONT]) or 1 of the 9 alternative forages at the inclusion level of 12% on a DM basis: Ms = *Medicago sativa*, Vs = *Vicia sativa*, Di = *Desmodium intortum*, Cc = *Calliandra calothyrsus*, Ll = *Leucaena leucocephala*, Ss = *Sesbania sesban*, Tz = *Tradescantia zebrina*, Ci = *Cichorium intybus*, or Ib = *Ipomoea batatas*.

2 Amount of CH_4_ produced per unit of digested organic matter (dOM).

3 Both OMD and ME were estimated from equations by Menke and Steingass (1988).

These reductions are consistent with in vivo findings (Cieslak et al., 2013; Ku-Vera et al., 2020), where feeding *Leucaena leucocephala* at up to 35% of dietary DM to cattle reduced CH_4_ emissions by 14% to 20%. Responses referred to in the studies were attributed mainly to CT and, potentially, to mimosine. However, pinpointing specific phytochemical drivers remains challenging due to the complex interactions among multiple compounds. In this study, despite similar CH_4_ reductions, Cc and Ll did not differ either in OMD or gas production compared with CON (*P* ≥ 0.05).

*Sesbania sesban* did not reduce CH_4_ production compared with CONT (*P* ≥ 0.05), contrasting with a 37% reduction reported when this legume was incubated with low-quality *Brachiaria humidicola* at a 0.20:0.80 ratio (Zeleke et al., 2006). The CH_4_:CO_2_ ratios were similar across treatments (*P* = 0.12). The CH_4_ per digested OM was lowest for Ll and Cc (40.7 and 41.5 mL/200 mg) and highest for CONT (45.4; *P* < 0.01), confirming their potential for practical mitigation.

Fermentation pH varied slightly among treatments, ranging from 6.89 in Tz to 7.08 in Ms (*P* < 0.01), but generally remained near-neutral, with only small reductions in some feed mixtures compared with CONT. Total VFA concentrations ranged from 45.8 m*M* in Vs to 49.4 m*M* in Ci (*P* < 0.01). Acetate, propionate, and butyrate molar proportions differed among treatments (*P* < 0.01), with acetate ranging from 69.1% (Tz) to 72.5% (Vs), propionate from 16.9% (Ci) to 19.0% (Tz), and butyrate from 7.05% (Ll) to 8.50% (Tz). Although CH_4_ production and acetate-to-propionate (**A:P**) ratio did not differ between CONT and Tz (*P* ≥ 0.05), the higher propionate in Tz (19.0%) suggests a greater glucogenic potential, consistent with its lower A:P ratio (3.71). Propionate redirects reducing equivalents away from methanogenesis and can improve energy efficiency and reduce CH_4_ emissions in vivo (Nozière et al., 2011). The A:P ratio differed among feed mixtures and ranged from 3.71 in Tz to 4.27 in Vs (*P* < 0.01).

Isovalerate (3-methylbutyric acid) was greatest in Ll (3.77%), more than 3-fold higher than CONT (*P* < 0.01), indicating leucine deamination (Bergman, 1990). *Vicia sativa* had the lowest total VFA concentration and a reduced isobutyrate (−44% vs. CONT; *P* < 0.01). Contrary to Garrett et al. (2021), Ci at 12% DM did not increase microbial protein production, based on NH_3_ and branched-chain VFA profiles (*P* ≥ 0.05). Ammonia was lowest in Cc and Ll (10.5 m*M*) and highest in Tz (11.7 m*M*; *P* < 0.01). Feed mixtures including temperate legumes (Ms, Vs) produced slightly higher NH_3_ (+ 0.55 m*M*) and CP (+ 0.95%) than tropical legumes (Di, Cc, Ll, and Ss). The elevated NH_3_ and propionate in Tz may warrant further investigation. The OMD and ME were higher for feed mixtures containing temperate legumes (Ms, Vs) than tropical legumes (Di, Cc, Ll, Ss; *P* < 0.01). The OMD ranged from 58.6% (Cc) to 62.5% (Ss), and ME ranged from 8.33 MJ/kg DM (Cc) to 8.72 MJ/kg DM (Ss). Mean ME (8.52 ± 0.14 MJ/kg DM) was 1.51 MJ/kg DM lower than values reported for higher-CP (16%) diets based on *Panicum maximum cv. Mombasa* (Meteab et al., 2025), likely reflecting the lower ME of the evaluated forages in this study.

The full GAM model explained 85.9% of the deviance in in vitro CH_4_ (adjusted R^2^ = 0.84), the cow variance component was 0.141 and the residual variance 0.039, whereas in the auxiliary “cow-only” and “feed mixture-only” GAM gave cow:residual variances of 0.203:0.104 and 0:0.137, respectively, indicating appreciable between-cow variation beyond incubated substrate effects. Hydrolyzable tannins showed no association with CH_4_ (Figure 1A: *k* = 1.00; *P* = 0.15), whereas CT showed a curvilinear negative trend (Figure 1B: *k* = 1.67; *P* = 0.06). Crude protein, NDF, and EE did not affect CH_4_ (Figure 1C, 1D, 1F; *P* ≥ 0.11). In the GAM models, each continuous predictor was represented by a smooth term with basis dimension *k*; an estimated effective df of 1 (*k* = 1) indicates an approximately linear relationship between the predictor and the response, whereas effective df values greater than 1 (*k* > 1) indicate increasing curvature. Increased dietary CP is often associated with modest reductions of CH_4_ via enhancing microbial protein synthesis that competes for hydrogen in rumen conditions (Hristov et al., 2013). The lack of effect of NDF was likely due to a narrow fiber range of the feed mixtures (from 51.9% to 58.1%), whereas the low EE levels (1.36%–1.73% of DM) were probably below levels required to inhibit methanogenesis, as fat supplementation in dairy cattle diets requires an average of 6.4% EE compared with control diets (2.5% EE; Eugène et al., 2008). In contrast, starch was negatively associated with CH_4_ production (Figure 1E; *P* < 0.01), consistent with hydrogen redirection toward propionate production formation instead of CH_4_ yield (Czerkawski, 1986). Conversely, OMD was positively associated with CH_4_ production (Figure 1G; *P* < 0.01), reflecting that diets with greater fermentability produce more substrate for methanogenesis (France and Dijkstra, 2005).

**Figure 1:**
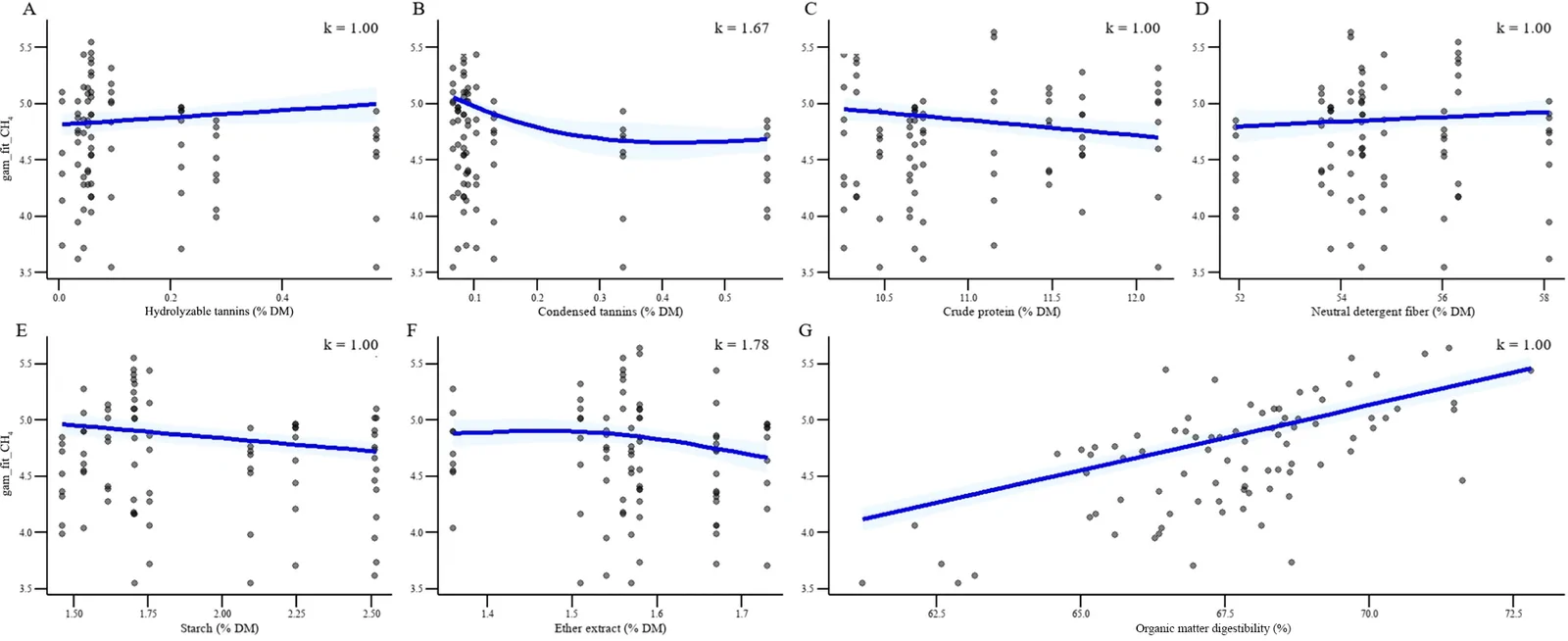
Response curves from the full generalized additive mixed model (GAM). Model-predicted relative change in CH_4_ production per 200 mg of DM (gam_fit_CH_4_) to (A) hydrolyzable tannins (*P* = 0.36); (B) condensed tannins (*P* = 0.06); (C) CP (*P* = 0.11); (D) NDF (*P* = 0.55); (E) starch (*P* < 0.01); (F) ether extract (*P* = 0.25); and (G) OM digestibility (*P* < 0.01). Shaded regions represent the prediction intervals calculated as fit ± SE at each value of x.

Tannins can inhibit methanogens by binding proteins and carbohydrates, reducing fiber degradation and promoting rumen fermentation toward propionate (Patra and Saxena, 2011). The present findings reinforce global evidence that CT, even at low inclusion levels typical of semi-intensive systems, can moderately reduce CH_4_ without inhibiting rumen fermentation or digestibility (Jayanegara et al., 2012). Overall, the GAM results support the direct CH_4_ reductions observed for Cc and Ll in vitro at 12% of DM inclusion, aligning with earlier in vivo studies with increased levels of supplementation (Molina et al., 2016; Mwangi et al., 2024).

Combined with their practical availability and low cost in East African systems, the tannin-containing shrubs evaluated here may provide scalable mitigation potential in Kenyan semi-intensive zero-grazing dairy systems. This study was limited by the use of rumen fluid from donor cattle fed diets not fully identical to the incubation substrates, although their overall nutrient composition was comparable. Future work should align diets more closely with the incubated substrate and include in vivo evaluations to confirm efficacy under practical feeding conditions, addressing aspects such as palatability, intake regulation, and long-term animal responses.
